# Enhanced carotenoid accumulation in *Chloroccocum humicola* under controlled CO_2_ and light conditions

**DOI:** 10.1016/j.jgeb.2025.100619

**Published:** 2025-11-24

**Authors:** Chatchai Kunyawut, Idtisak Paopo, Chakkrit Umpuch

**Affiliations:** Department of Chemical Engineering, Ubon Ratchathani University, 85 Sathonlamark road, Warin Chamrap, Ubon Ratchathani 31490, Thailand

**Keywords:** Carotenoid production, Green microalgae, CO_2_ enrichment, Light intensity modulation, β-carotene, Astaxanthin accumulation

## Abstract

Carotenoids are potent antioxidants and high-value bioactive compounds that green microalgae can efficiently synthesize. This study aimed to enhance carotenoid production in *Chlorococcum humicola* TISTR 8551 using a two-stage cultivation strategy in a 10-L air-lift photobioreactor (ALPBR), separating biomass accumulation and stress induction phases. During the “green stage” (9 days), cells were grown in modified BG-11 medium (N:P ratio 31:1) under 3,500 Lux white LED light with varying CO_2_ concentrations (1–3 % v/v) to maximize biomass yield. In the subsequent “red stage” (15 days), environmental stressors, including elevated salinity and intensified light exposure, were applied to stimulate carotenoid biosynthesis. The highest total carotenoid content (38.72 ± 1.04 mg/L, 0.313 ± 0.018 mg/g biomass) was observed under 3 % CO_2_ supplementation, likely due to enhanced photosynthetic carbon fixation and improved precursor availability via glucose metabolism. An optimal white light intensity of 25,000 Lux produced 32.03 ± 1.52 mg/L carotenoids. Additionally, the combination of 100,000 Lux white light with 1,600 Lux blue light significantly increased β-carotene content (6.98 ± 0.28 % of total carotenoids), while 2,400 Lux blue light yielded the highest astaxanthin level (5.01 ± 0.18 % of total carotenoids). These results highlight the synergistic effects of CO_2_ enrichment**,** spectral light modulation**,** and stage-specific stress application in promoting targeted carotenoid biosynthesis. This study offers practical insights for optimizing large-scale microalgal pigment production in controlled photobioreactor systems.

## Highlights

• Two-stage cultivation boosted carotenoid yield in Chlorococcum humicola.
• CO_2_ enrichment at 3 % enhanced β-carotene and astaxanthin levels.
• White light at 25,000 Lux promoted high carotenoid productivity.
• Blue light (1600–2400 Lux) stimulated secondary carotenoid synthesis.
• Strategy offers a scalable method for microalgal pigment production.

## Graphical abstract

**Figure:**
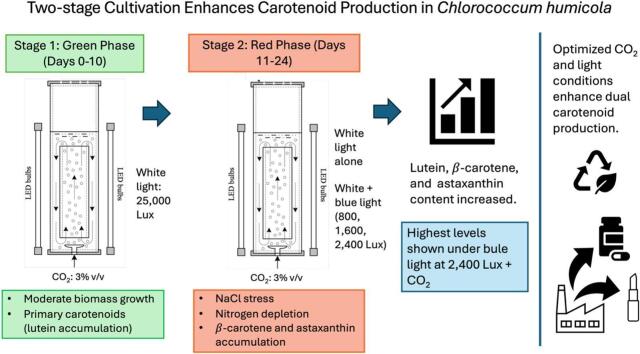

## 1 Introduction

Carotenoids are a structurally diverse group of lipophilic pigments that play indispensable roles in photosynthesis and photoprotection. They stabilize thylakoid membranes, broaden light absorption, and mitigate oxidative damage via non-photochemical quenching. Beyond their physiological functions, carotenoids are of high commercial value due to their antioxidant properties and applications in food, nutraceutical, cosmetic, and aquaculture industries.^1^ Structurally, they are classified into two major groups: carotenes (e.g., β-carotene) and xanthophylls (e.g., lutein, zeaxanthin, astaxanthin).^2^, ^3^ Globally, six major carotenoids—β-carotene, lutein, astaxanthin, zeaxanthin, lycopene, and canthaxanthin—dominate the market, with β-carotene, lutein, and astaxanthin together accounting for more than half of total sales.^4^

Microalgae are increasingly recognized as superior platforms for carotenoid production due to their rapid growth, high areal productivity, and ability to accumulate pigments under stress. Well-studied strains include *Dunaliella salina*, *Haematococcus pluvialis*, and *Chlorella* spp., which serve as benchmarks for industrial-scale pigment production.^5^ *D. salina* hyperaccumulates β-carotene under hypersaline conditions, *H. pluvialis* produces large amounts of astaxanthin during encystment, and *Chlorella* spp. are cultivated widely for lutein and other xanthophylls. While these models have an advanced understanding of algal photophysiology, they often require extreme stress conditions or complex life-cycle transitions, limiting industrial scalability.^6^

In contrast, *Chlorococcum humicola* exhibits unique physiological traits that underscore its novelty as a carotenoid-producing species. Unlike *D. salina* and *H. pluvialis*, which require hypersalinity or cyst formation, *C. humicola* can accumulate substantial levels of lutein and β-carotene under milder stress, with reported yields up to 20 mg/g biomass.^7^, ^8^, ^9^ Its tolerance to moderate stress, ability to maintain prolonged stationary growth, and capacity for pigment production without severe environmental manipulation simplify cultivation and reduce operational costs. These traits position *C. humicola* as a promising candidate for carotenoid biosynthesis, particularly in photobioreactor systems where environmental parameters can be optimized without reliance on extreme conditions.

Despite these advantages, the physiology of *C. humicola* remains underexplored compared with established industrial strains. Most previous studies have been restricted to open ponds or uncontrolled laboratory setups, limiting reproducibility and scalability.^10^ Moreover, systematic investigations into the combined influence of CO_2_ enrichment and light spectral quality on carotenoid accumulation in *C. humicola* are lacking.^11^ Addressing these knowledge gaps is critical to establishing its potential as an industrial strain distinct from existing models.

This study advances current knowledge by examining how CO_2_ concentration and variations in white and blue light intensity affect biomass production and carotenoid accumulation in *C. humicola*. By applying a two-stage cultivation strategy in an air-lift photobioreactor (ALPBR), we demonstrate how mild, controllable stressors can be leveraged to enhance pigment biosynthesis in this species. The findings highlight the physiological advantages of *C. humicola* and clarify its potential to complement or even outperform traditional strains under scalable, energy-efficient cultivation systems.

## 2 Materials and methods

### 2.1 Microalgae strain and culture medium

The freshwater green microalga *Chlorococcum humicola* TISTR 8551 was obtained from the Thailand Institute of Scientific and Technological Research (TISTR). The strain was cultivated in a modified BG-11 medium,^12^ comprising macronutrients (1,500 mg/L NaNO_3_, 40 mg/L K_2_HPO_4_·3H_2_O, 75 mg/L MgSO_4_·7H_2_O, 36 mg/L CaCl_2_·2H_2_O, and 20 mg/L Na_2_CO_3_) and micronutrients (6 mg/L citric acid, 6 mg/L ferric ammonium citrate, and 1 mg/L EDTA). A trace metal solution (TMA5) was supplemented at 1.0 mL/L, consisting of 2,860 mg/L H_3_BO_3_, 1,810 mg/L MnCl_2_·4H_2_O, 390 mg/L Na_2_MoO_4_·2H_2_O, 222 mg/L ZnSO_4_·7H_2_O, 79 mg/L CuSO_4_·5H_2_O, and 50 mg/L Co(NO_3_)_2_·6H_2_O. To induce nitrogen-limited conditions, the concentrations of NaNO_3_ and K_2_HPO_4_·3H_2_O were reduced to 150 mg/L and 13 mg/L, respectively, resulting in a nitrogen-to-phosphorus (N:P) molar ratio of 31:1. The culture medium was adjusted to pH 7.0 and sterilized by autoclaving at 120 °C for 20 min (Model GF-TR, ZEALWAY, USA).

### 2.2 Inoculum preparation

Axenic pre-cultures were grown in 500 mL Erlenmeyer flasks containing 300 mL of modified BG-11 medium and maintained at 30 ± 2 °C under continuous white LED illumination at 3,500 Lux. The pH was adjusted to 7.0 using either 1 M HCl or 1 M NaOH. Filtered air (0.45 µm) was supplied continuously for 14 days. Microalgal morphology was periodically monitored using a light microscope (Model: ECLIPSE Ei, Nikon, Japan) at 100× magnification. Following sufficient cell growth, cultures were transferred to the air-lift photobioreactor (ALPBR) for subsequent experiments.

### 2.3 Air-Lift Photobioreactor (ALPBR) setup

**Fig. 1:**
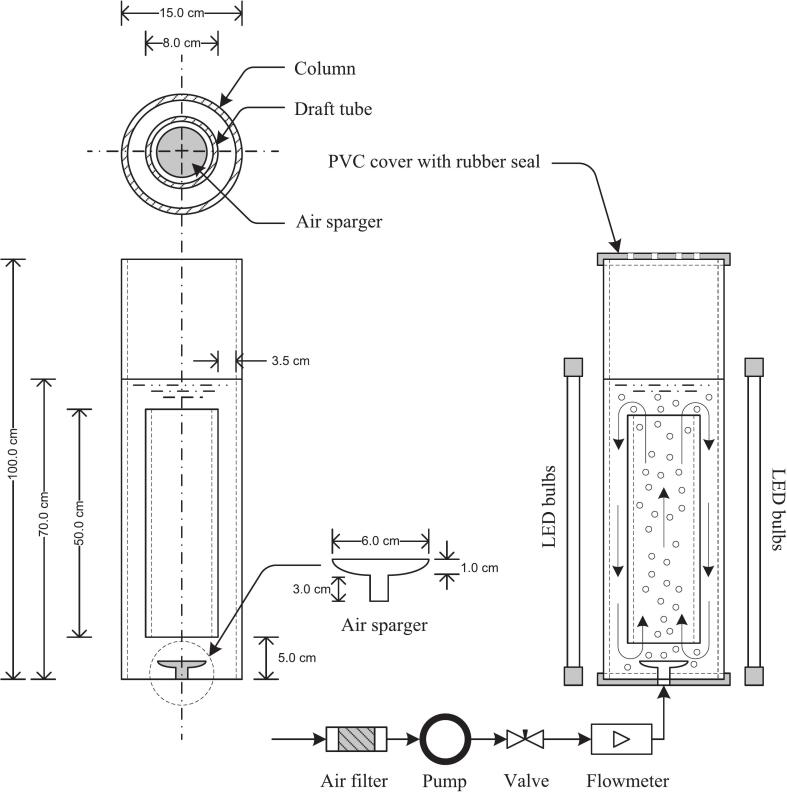
ALPBR and accessories (Not to scale).

A 10-L Cylindrical Air-Lift Photobioreactor (ALPBR), shown in Fig. 1, was constructed using transparent acrylic tubing with an outer diameter of 15 cm and a height of 100 cm. The internal draft tube measured 8 cm in diameter and 50 cm in height, resulting in an overall aspect ratio of 2.62.^13^ Aeration was provided through a custom-fabricated flat plate sparger at a superficial velocity of 0.3–0.5 cm/s. To maintain sterility, compressed air was passed through a 0.45 µm syringe filter (Minisart SRP15, Sartorius, Germany). Illumination was supplied by high-intensity white LED panels (HL-WP100, HiLight Technology Co., Ltd., Thailand) mounted externally around the reactor. For specific treatments, supplemental blue LED modules (BL-450NM, OptoLED, Taiwan) were employed. Light intensity was quantified using a digital lux meter (TES-1330A, TES Electrical Electronic Corp., Taiwan). The reactor body was fabricated from food-grade PVC and polyacrylic materials to ensure structural durability and biosafety.

### 2.4 Optimization of initial microalgae cell density

To determine the optimal initial optical density (OD), batch cultures of *Chlorococcum humicola* were inoculated at OD_680_ values of 0.1, 0.3, and 0.5, and cultivated for 14 days under standard conditions (3500 Lux white LED light, 28–32 °C, 12:12 h light/dark cycle, pH 7.0, and aeration at 0.5 cm/s). Daily 50 mL samples were collected to measure cell density, dry biomass, residual nitrate and phosphate, chlorophyll, fatty acids, and total carotenoids.

The specific growth rate (*μ*, day^−1^) and specific productivity (SP, mg/L/day) were calculated using Eqs. (1), (2):

(1) $$\mu =\frac{\ln N_{2}-{\ln N}_{1}}{t_{2}-t_{1}}$$

(2) $$\mathrm{SP}=\frac{X_{2}-X_{1}}{t_{2}-t_{1}}$$

where *N*_1_ and *N*_2_ are cell densities (cells/mL) at time *t*_1_ and *t*_2_ (day), respectively, and *X*_1_ and *X*_2_ represent biomass concentrations (mg/L) at the same time points. Eq. (2) was also applied to calculate the specific productivities of biochemical compounds, including fatty acids, chlorophyll, and total carotenoids, by substituting their concentrations for *X*.

This standardized approach allowed consistent evaluation of growth performance and metabolite productivity across different inoculum densities.

### 2.5 Effect of CO_2_ concentration

To evaluate the influence of CO_2_ enrichment on growth and carotenoid biosynthesis, *C. humicola* was cultivated under 1 %, 2 %, and 3 % (v/v) CO_2_ during the light phase (12 h/day) of the green stage (days 0–9). In the subsequent red stage (days 10–24), CO_2_ supplementation was discontinued, and salinity stress was applied by sequential addition of NaCl (100 mM every 5 days, up to 300 mM). White LED light intensity was maintained at 3,500 Lux for the entire cultivation period.

The selected CO_2_ concentrations (1–3 % v/v) were determined based on both literature evidence and preliminary optimization trials. Previous studies reported that low to moderate enrichment (≤3 %) enhances biomass productivity and metabolic activity in several green microalgae species, while higher concentrations typically induce medium acidification, CO_2_ oversaturation, and impaired photosynthetic efficiency.^14^, ^15^ In our preliminary experiments (data not shown), supplementation above 3 % resulted in reduced growth performance, thereby supporting the decision to restrict the tested range to 1–3 % CO_2_ as physiologically relevant and non-inhibitory.

Importantly, CO_2_ supplementation was discontinued during the red stage to avoid the detrimental effects of excessive acidification observed under prolonged CO_2_ injection. Preliminary trials demonstrated that continued CO_2_ supply during the stress phase substantially lowered culture pH, leading to sharp reductions in cell viability and biomass. To impose stress without compromising survival, salinity stress was applied as an alternative trigger for carotenoid biosynthesis. Stepwise NaCl addition (up to 300 mM) provided osmotic and ionic stress that effectively induced carotenoid accumulation, while avoiding the compounding impact of CO_2_-induced medium acidification. This decoupling of CO_2_ and salinity stress allowed us to sustain culture viability during the red stage while still enhancing pigment biosynthesis, aligning with the physiological goals of stress-induced secondary metabolite production.

### 2.6 Effect of white and blue light intensity

To investigate the influence of irradiance, white LED light intensities ranging from 3,500 to 100,000 Lux were applied, simulating conditions from shaded environments to full sunlight. These levels were selected to encompass the thresholds of light saturation and photoinhibition reported in previous studies. During the red stage, white light intensities between 12,500 and 100,000 Lux were tested to evaluate their impact on carotenoid accumulation.

In addition, the effect of blue light on pigment biosynthesis was examined by supplementing 100,000 Lux white light with blue LED illumination at 800, 1,600, and 2,400 Lux, resulting in combined intensities of 100,800–102,400 Lux. These intensities were chosen based on prior evidence that blue light enhances chlorophyll and carotenoid synthesis in green microalgae. Culture temperature was maintained at 35 ± 1.5 °C, and cultures were monitored daily to assess growth, biomass, pigment accumulation, and nutrient consumption under each condition.

### 2.7 Analytical methods

Cell density was determined by measuring the optical density (OD) at 680 nm using a UV–visible spectrophotometer (Model: UV-1800, Shimadzu Corp., Japan). A calibration curve was generated by correlating OD_680_ values with cell counts obtained using a hemocytometer (Neubauer Improved, Marienfeld, Germany) following standard procedures.^16^ Dry cell weight (DCW, mg/L) was measured by filtering 50 mL of culture through pre-weighed 0.45 µm GF/C filters (Whatman™, USA), drying at 105 °C for 24 h, and reweighing.^17^

Nitrate concentration was analyzed using the UV screening method according to APHA Standard Methods (Method 4500-NO_3_^−^), with absorbance measured at 220 and 275 nm and corrections for organic interference.^18^ Phosphate concentration was determined using the molybdenum blue method, where ammonium molybdate and ascorbic acid reagents reacted with filtered samples, and absorbance was measured at 880 nm.^19^

Chlorophyll and total carotenoids were extracted from 5 mL of culture using 5 mL of 95 % ethanol. The pellet was incubated at 65 °C for 15 min, then centrifuged using a centrifuge (Model: 225(LXJ12), Fisher Scientific Centrific, USA). The supernatant was analyzed spectrophotometrically at 470, 645, and 663 nm. The concentrations of total chlorophyll and carotenoids were calculated using the Lichtenthaler and Wellburn equations.^20^

Fatty acids were extracted following the Bligh and Dyer method.^21^ Concentrated biomass was extracted with chloroform:methanol:water (1:2:0.8, v/v/v), and the organic phase was collected. Lipids were transmethylated with 2 % sulfuric acid in methanol at 80 °C for 2 h to form fatty acid methyl esters (FAMEs), which were analyzed by gas chromatography (Model: GC-2014, Shimadzu, Japan) equipped with a flame ionization detector (FID) and a DB-WAX column (30 m × 0.25 mm, 0.25 µm).

Individual carotenoids were quantified by high-performance liquid chromatography (HPLC). Freeze-dried biomass (10 mg) was extracted with 5 mL acetone, filtered through a 0.45 µm PTFE membrane, and injected into an HPLC system (Model: LC-20AT, Shimadzu Corp., Japan) equipped with a C18 column (Shim-pack GIST, 4.6 × 250 mm, 5 µm). The mobile phase was methanol:acetonitrile:dichloromethane (70:20:10, v/v/v) at a flow rate of 1.0 mL/min, with detection at 450 nm. Carotenoids were identified and quantified by comparison with authentic standards (Sigma-Aldrich, USA).^22^, ^23^

### 2.8 Statistical analysis

All experiments were performed in triplicate, and results were reported as mean ± standard deviation (SD). One-way analysis of variance (ANOVA) was used to evaluate differences among treatments. When significant effects were detected (p < 0.05), Tukey’s honestly significant difference (HSD) test was applied for post hoc multiple comparisons. For targeted pairwise comparisons between control and treatment groups, independent-samples t-tests were conducted. Prior to conducting ANOVA, normality and homogeneity of variances were tested using the Shapiro–Wilk and Levene’s tests, respectively, and all datasets met these assumptions (p > 0.05). Effect sizes (η^2^) were calculated to evaluate the practical relevance of the findings. Statistical significance was set at α = 0.05. All analyses were conducted using Minitab version 19.0. Data in tables were expressed as mean ± SD to indicate variability among replicates, whereas figures displayed mean ± standard error (SE) to illustrate the precision of the mean and facilitate treatment comparisons.

## 3 Results and discussion

### 3.1 Optimization of initial cell density for biomass and carotenoid production

#### 3.1.1 Growth kinetics and biomass formation

**Fig. 2:**
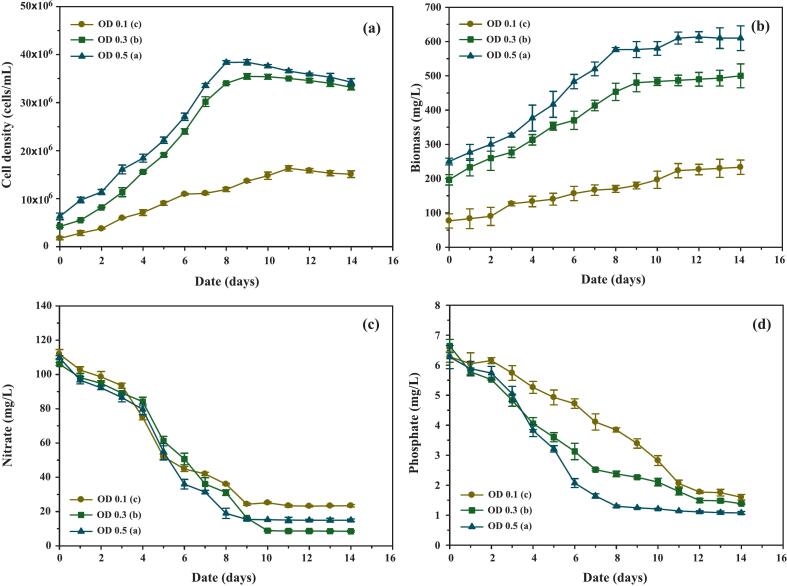
Growth performance, biomass accumulation, and nutrient uptake of *C. humicola* cultivated under different initial optical densities (OD_680_ 0.1, 0.3, and 0.5) over a 14-day cultivation period. Parameters include: (a) cell density (cells/mL), (b) biomass concentration (mg/L), (c) nitrate concentration (mg/L), and (d) phosphate concentration (mg/L). Data are presented as mean ± SE (n = 3). Different lowercase letters (a–c) indicate statistically significant differences among treatments based on Tukey’s HSD test (p < 0.05).

The growth performance of *C. humicola* was evaluated at initial OD_680_ values of 0.1, 0.3, and 0.5 over a 14-day batch cultivation. As shown in Fig. 2a, all treatments exhibited the typical microbial growth profile, characterized by exponential, stationary, and decline phases. No lag phase was observed under any condition, likely because the inoculum was pre-adapted to the cultivation medium with a consistent N:P molar ratio of 31:1.

The culture initiated at an OD_680_ of 0.3 achieved a maximum cell density of 35.50 × 10⁶ ± 0.57 × 10⁶ cells/mL by day 9, slightly below that of the OD_680_ 0.5 culture (38.40 × 10⁶ ± 0.38 × 10⁶ cells/mL). However, OD_680_ 0.3 stabilized earlier and exhibited less pronounced cell death, suggesting a more favorable balance between growth and survival. Biomass trends (Fig. 2b) closely mirrored cell density, with the exception of the OD_680_ 0.1 treatment, where biomass continued to rise despite declining viable cell counts. This discrepancy indicates that total biomass measurements included both viable and non-viable cells, whereas cell density reflected only metabolically active populations.

Specific growth rates (*μ*) during the exponential phase were 0.229 ± 0.025, 0.237 ± 0.026, and 0.201 ± 0.022 day^−1^ for initial OD_680_ values of 0.1, 0.3, and 0.5, respectively. These results suggest that OD_680_ 0.3 offered the most favorable growth kinetics, balancing rapid biomass accumulation with minimal self-shading. This trend is consistent with the findings of Slocombe et al.,^24^ who demonstrated optimal biomass productivity at intermediate inoculum densities in *Chlorella vulgaris*. Similarly, Ahmed et al.^25^ reported that excessively high inoculum densities reduced light penetration and photosynthetic efficiency, leading to lower pigment accumulation—a phenomenon also evident in our OD_680_ 0.5 cultures.

#### 3.1.2 Nutrient utilization and conversion efficiency

As shown in Fig. 2c-d, nitrate and phosphate concentrations declined markedly during the exponential phase across all treatments. The highest absolute consumption rates were observed in the OD_680_ 0.5 culture, reflecting its greater metabolic demand due to higher cell density. However, when normalized to biomass, nitrate utilization efficiency was notably higher in the OD_680_ 0.3 culture (0.35 ± 0.04 mg/L/mg biomass) compared to OD_680_ 0.5 (0.26 ± 0.03 mg/L/mg biomass), indicating more efficient nitrogen assimilation at the intermediate inoculum level.

Phosphate consumption followed a similar pattern, with OD_680_ 0.3 showing moderate absolute uptake but superior utilization efficiency relative to OD_680_ 0.5. The N:P molar ratio at day 9 was 11.08:1 for OD_680_ 0.3, which falls within the optimal range reported by Wang et al.^26^ for balanced algal growth and carotenoid biosynthesis. This favorable stoichiometric balance likely enhanced intracellular carbon flux toward secondary metabolite pathways, consistent with the higher pigment accumulation observed in subsequent analyses.

Collectively, these findings indicate that an initial OD_680_ of 0.3 represents an optimal compromise between rapid biomass growth and efficient nutrient utilization. This balance provides a robust physiological foundation for maximizing carotenoid biosynthesis during the stress-induced red stage.

#### 3.1.3 Metabolite Accumulation: Fatty acid, Chlorophyll, and carotenoids

**Fig. 3:**
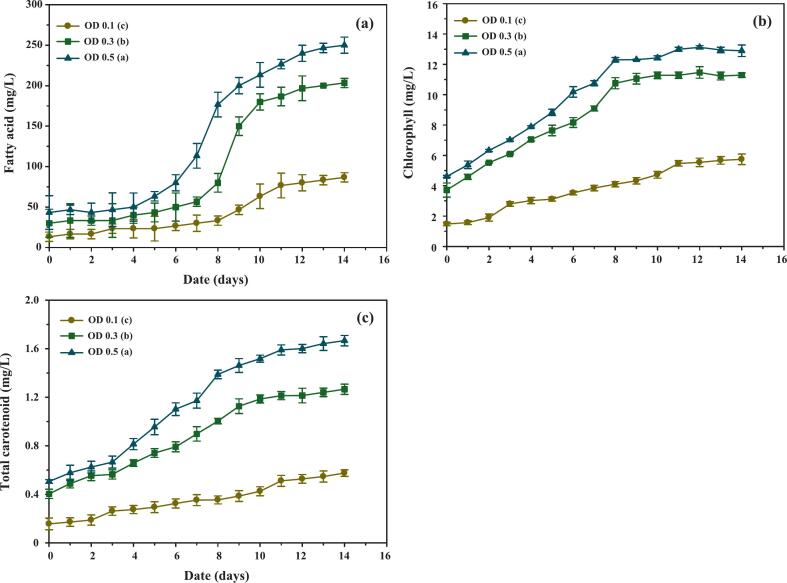
Accumulation of intracellular components in *C. humicola* cultivated under different initial optical densities (OD 0.1, 0.3, and 0.5) during a 14-day cultivation period. The measured parameters include: (a) fatty acid concentration (mg/L), (b) chlorophyll concentration (mg/L), and (c) total carotenoid concentration (mg/L). Data are presented as mean ± SE (n = 3). Different lowercase letters (a–c) denote statistically significant differences among treatments based on Tukey’s HSD test (p < 0.05).

Fatty acid accumulation patterns (Fig. 3a) showed an initial gradual increase across all treatments during the first five days, reflecting adequate nitrogen availability. A sharp rise occurred between days 5 and 8, particularly in cultures initiated at OD_680_ 0.3 and 0.5, coinciding with nitrate depletion and signaling a metabolic redirection toward lipid biosynthesis. After day 8, accumulation slowed, likely due to nutrient exhaustion, the onset of the stationary phase, and increasing cell mortality. As reported in Table 1, OD_680_ 0.3 achieved significantly higher fatty acid productivity than OD_680_ 0.1, while remaining comparable to OD_680_ 0.5, suggesting that intermediate inoculum density enables efficient lipid synthesis without the drawbacks of excessive crowding. This observation aligns with prior studies in *Chlorella* and *Scenedesmus*, where nitrogen stress was shown to drive lipid accumulation as a carbon sink.^27^

**Table 1:** Effect of initial optical density on bioproducts accumulation in *C. humicola* during 14-day cultivation. Data are presented as mean ± SD (n = 3). Different superscript letters indicate significant differences among treatments (*p* < 0.05, Tukey’s HSD test). One-way ANOVA revealed significant effects on total carotenoid production (F[2,132] = 75.3, *p* < 0.001).

Initial OD	0.1	0.3	0.5
Biomass (mg/L)	2,433.30 ± 88.88^c^	5,803.30 ± 100.79^b^	7,126.70 ± 102.83^a^
Specific biomass (mg/L/day)	173.80 ± 7.13^c^	414.52 ± 32.10^b^	509.00 ± 32.75^a^
Fatty acid (mg/L)	643.33 ± 41.19^c^	1,460.00 ± 55.73^b^	2,040.00 ± 57.14^a^
Specific fatty acid (mg/L/day)	45.95 ± 2.30^c^	104.29 ± 5.22^b^	145.71 ± 7.28^a^
Chlorophyll (mg/L)	56.84 ± 1.27^c^	130.23 ± 4.42^b^	149.98 ± 3.55^a^
Specific chlorophyll (mg/L/day)	4.06 ± 0.09^c^	9.30 ± 0.32^b^	10.71 ± 0.25^a^
Total carotenoid (mg/L)	5.35 ± 0.48^c^	13.34 ± 1.05^b^	17.28 ± 0.85^a^
Specific total carotenoid (mg/L/day)	0.382 ± 0.034^c^	0.953 ± 0.075^b^	1.234 ± 0.061^a^

a,b,c Different superscript letters within the same row indicate significant differences among treatments (p < 0.05, Tukey's HSD test).

Chlorophyll concentrations (Fig. 3b) rose steadily until day 9, consistent with active growth under nutrient-replete conditions. Beyond day 9, accumulation slowed as nitrate became limiting, especially in OD_680_ 0.1 cultures. Significantly higher chlorophyll content was observed at OD_680_ 0.3 and 0.5, confirming that initial biomass influences pigment biosynthesis and light-harvesting efficiency. This pattern is consistent with reports in *Scenedesmus obliquus*, where moderate inoculum densities enhanced pigment accumulation due to balanced light exposure and nutrient availability.^27^

Carotenoid levels (Fig. 3c) increased gradually throughout the cultivation period but remained approximately ninefold lower than chlorophyll concentrations, indicating that mild nitrogen limitation alone did not trigger substantial carotenoid induction under batch conditions. Nonetheless, OD_680_ 0.3 cultures achieved the highest specific carotenoid productivity (Table 1), representing an optimal balance between moderate stress and metabolic activity. These findings are in agreement with Han et al.,^28^ who reported enhanced carotenoid biosynthesis under moderate inoculum densities in *Haematococcus pluvialis*, and with results from *Scenedesmus* under semi-continuous cultivation.^29^

#### 3.1.4 Selection of optimal initial OD

At the late exponential phase (day 9), the N:P molar ratio was 11.08:1 in OD_680_ 0.3 cultures and 19.19:1 in OD_680_ 0.5 cultures. While the Redfield ratio (16:1) is widely regarded as optimal for balanced algal growth and pigment biosynthesis,^30^ the moderate nitrogen limitation at OD_680_ 0.3 appeared to stimulate carotenoid accumulation without compromising viability. In contrast, OD_680_ 0.5 maintained nitrogen sufficiency longer, which may have delayed the transition to secondary metabolite pathways and contributed to higher mortality after day 10.

Cultures initiated at OD_680_ 0.1 exhibited limited productivity due to insufficient starting biomass, while OD_680_ 0.5 suffered from reduced light penetration and shading effects—both of which have been extensively documented in dense algal systems.^31^ Previous studies with *Haematococcus pluvialis* and *Scenedesmus* sp. identified optimal inoculum densities in the range of OD_680_ 0.2–0.4 for maximizing carotenoid yields under two-stage cultivation.^32^ Our findings corroborate these reports, confirming that moderate inoculum levels provide the best balance between nutrient uptake, light availability, and carotenoid biosynthesis.

Statistical analyses reinforced these trends. One-way ANOVA revealed significant effects of initial optical density on carotenoid productivity (F[2,132] = 75.3, p < 0.001). Tukey’s post-hoc test indicated that OD_680_ 0.5 produced the highest absolute carotenoid yield (17.28 ± 0.85 mg/L), significantly greater than OD_680_ 0.3 (13.34 ± 1.05 mg/L, p < 0.01) and OD_680_ 0.1 (5.35 ± 0.48 mg/L, p < 0.001). However, when integrating considerations of growth stability, nutrient efficiency, and cell viability, OD_680_ 0.3 emerged as the most practical choice for subsequent red-stage induction, supporting robust and sustainable carotenoid productivity.

### 3.2 Effects of CO_2_ concentration on growth and carotenoid production

#### 3.2.1 Growth response and cell viability

**Fig. 4:**
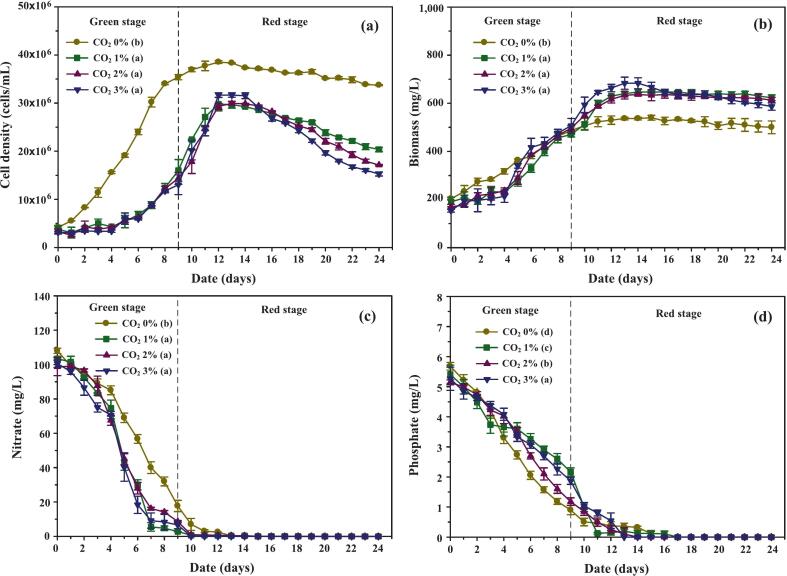
Effects of different CO_2_ concentrations (0 %, 1 %, 2 %, and 3 % v/v) on the growth performance, biomass accumulation, and nutrient uptake of *C. humicola* during the green stage (days 0–9). Measured parameters include: (a) cell density (cells/mL), (b) biomass concentration (mg/L), (c) nitrate concentration (mg/L), and (d) phosphate concentration (mg/L). Data are expressed as mean ± SE (n = 3). Different lowercase letters (a–d) indicate statistically significant differences among treatments according to Tukey’s HSD test (p < 0.05). The dashed vertical line indicates the transition point between the green and red cultivation stages.

As shown in Fig. 4a, *C. humicola* exhibited distinct growth dynamics under varying CO_2_ levels across the 24-day cultivation. During the green stage (days 0–9), control cultures (no CO_2_) achieved significantly higher cell densities, reaching 35.40 × 10⁶ ± 0.53 × 10⁶ cells/mL—approximately 2.2–2.7 times greater than CO_2_-enriched treatments. In contrast, CO_2_ supplementation induced a lag phase lasting four days, likely due to medium acidification (pH < 5), before exponential growth resumed. Although pH adjustments partially mitigated this stress, specific growth rates under 1–3 % CO_2_ remained ∼32 % lower than in controls, indicating that elevated carbon initially imposes physiological constraints. This response is consistent with findings in *Chlorella vulgaris* and *Scenedesmus obliquus*, where CO_2_ concentrations above 2 % suppress early growth but subsequently enhance metabolic flux once adaptation occurs.^33^, ^34^

During the red stage (days 10–24), the growth advantage shifted toward CO_2_-enriched cultures. While control cultures stagnated after day 13 and entered decline by day 15, CO_2_-supplemented treatments continued to grow, peaking at day 12 with 46–59 % increases over day 9 values before gradually declining. The delayed decline reflects greater resilience to stress, although higher CO_2_ concentrations were associated with sharper decreases by day 24. This suggests that prior CO_2_ exposure enhanced metabolic capacity but also exacerbated late-stage mortality when salinity stress intensified.

The combined effects of CO_2_ and salinity stress underscore the delicate balance between stimulating secondary metabolism and maintaining viability. Moderate NaCl additions initially promoted growth and metabolite accumulation, but concentrations above 200 mM accelerated mortality. Elevated salinity increased osmotic pressure and triggered reactive oxygen species (ROS) formation, particularly hydroxyl radicals (•OH), which in turn activated calcium influx pathways leading to fatty acid and carotenoid biosynthesis as protective responses.^35^ Similar adaptive mechanisms have been reported by Ho et al.,^36^ where mild CO_2_ stress enhanced resistance to subsequent oxidative and osmotic stress via upregulation of antioxidant enzymes and membrane-stabilizing metabolites.

Overall, these findings highlight that CO_2_ enrichment initially hinders growth through acid stress but later facilitates improved stress tolerance and secondary metabolism. Effective cultivation strategies for *C. humicola* must therefore balance CO_2_ availability and salinity to prolong viability, enhance carbon flux, and optimize carotenoid biosynthesis under multi-phase cultivation systems.

#### 3.2.2 Nutrient uptake and stress response

As shown in Fig. 4c, nitrate depletion patterns revealed distinct differences between CO_2_-enriched and control cultures. During the first four days, nitrate consumption was similar across all treatments, reflecting comparable metabolic demand during early adaptation. However, from day 5 onwards, nitrate declined more sharply in CO_2_-supplemented cultures, with residual levels at day 9 reduced to just 2.77 %, 8.73 %, and 6.61 % of the initial concentration under 1.0 %, 2.0 %, and 3.0 % CO_2_, respectively, compared to 16.41 % in the control. This indicates that although elevated CO_2_ initially delayed cell proliferation (Fig. 4a), once adaptation occurred, nitrogen assimilation accelerated. The likely explanation is that improved carbon flux from CO_2_ fixation supported amino acid and pigment synthesis, thereby elevating nitrogen demand during the exponential recovery phase.^37^

Phosphate utilization exhibited a complementary trend (Fig. 4d). Early in cultivation (days 0–4), phosphate consumption was minimal, especially in CO_2_-enriched cultures where growth lagged. From days 5–9, however, the control group showed a marked reduction in phosphate, while CO_2_-supplemented treatments consumed phosphate more gradually, reflecting delayed cell division. By day 15, phosphate levels had dropped to nearly undetectable concentrations in all conditions, signaling the complete exhaustion of macronutrients during the red stage. This nutrient exhaustion coincided with the onset of secondary metabolism, suggesting that nutrient depletion served as a critical trigger for carotenoid biosynthesis.^38^

The enhanced nutrient removal observed under CO_2_ enrichment is consistent with previous findings in *Haematococcus pluvialis* and *Botryococcus braunii*, where elevated CO_2_ improved macromolecular synthesis and stimulated nutrient turnover under stress.^37^, ^38^ Together, these results suggest that *C. humicola*, once acclimated to CO_2_-induced acid stress, reprograms its metabolism toward more efficient nitrate and phosphate assimilation. This metabolic reconfiguration provides the necessary precursors for secondary metabolite pathways, facilitating enhanced carotenoid accumulation during the red stage.

#### 3.2.3 Pigment and metabolite accumulation

**Fig. 5:**
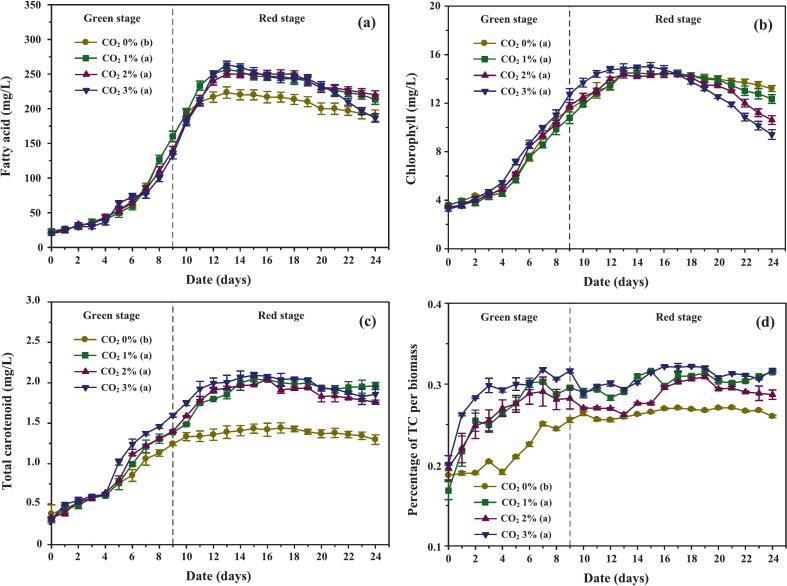
Accumulation of intracellular biomolecules and carotenoids in *C. humicola* cultivated under different CO_2_ concentrations (0 %, 1 %, 2 %, and 3 % v/v) during the green stage (days 0–9). Parameters include: (a) fatty acid concentration (mg/L), (b) chlorophyll concentration (mg/L), (c) total carotenoid concentration (mg/L), and (d) total carotenoids as a percentage of biomass. Data are presented as mean ± SE (n = 3). Different lowercase letters (a–b) indicate statistically significant differences among treatments based on Tukey’s HSD test (p < 0.05). The dashed vertical line represents the transition between the green and red cultivation stages.

Fig. 5a illustrates fatty acid dynamics under CO_2_-enriched and control conditions. During the green stage, fatty acid levels rose gradually and remained similar across treatments, despite the lower cell densities observed under CO_2_ supplementation (Fig. 4a). This paradox can be explained by larger average cell size and higher dry biomass in CO_2_-treated cultures, a pattern also noted in *Chlorella* sp..^39^ In the red stage, fatty acid levels increased more prominently in CO_2_-enriched treatments, reflecting stress responses triggered by nitrogen depletion and osmotic pressure. Similar shifts in metabolism have been documented in *Scenedesmus obliquus*, where chloride ions interfered with NADPH regeneration and redirected carbon toward lipid and carotenoid synthesis.^40^ Beyond day 13, fatty acid concentrations declined in all treatments, likely due to nutrient exhaustion and cell death. Despite comparable final biomass yields, cumulative fatty acid productivity under CO_2_ enrichment improved by ∼8.8 % (Table 2), highlighting the stimulatory effect of CO_2_ on stress-driven metabolite allocation.

**Table 2:** Effect of CO_2_ concentration on bioproducts accumulation in *C. humicola* during 24-day cultivation, with CO_2_ supplementation stopped after day 10. Data are expressed as mean ± SD (n = 3). Different superscript letters denote significant differences among CO_2_ treatments at p < 0.05 according to Tukey’s HSD test. One-way ANOVA revealed a highly significant effect on total carotenoid production (F[3,176] = 177.6, p < 0.001).

CO_2_ concentration (%v/v)	0 (Air only)	1	2	3
Biomass (mg/L)	11,246.67 ± 72.82^b^	12,396.00 ± 70.15^a^	12,370.00 ± 64.18^a^	12,653.33 ± 71.60^a^
Specific biomass (mg/L/day)	468.61 ± 14.89^b^	516.53 ± 14.34^a^	515.42 ± 13.12^a^	527.22 ± 20.50^a^
Fatty acid (mg/L)	3,770.00 ± 56.55^b^	4,160.00 ± 62.40^a^	4,113.33 ± 61.70^a^	4,033.33 ± 50.41^a^
Specific fatty acid (mg/L/day)	157.08 ± 2.53^b^	173.33 ± 2.12^a^	171.39 ± 1.98^a^	168.06 ± 1.66^a^
Chlorophyll (mg/L)	271.28 ± 4.74^a^	266.94 ± 4.84^a^	267.62 ± 4.11^a^	268.88 ± 5.00^a^
Specific chlorophyll (mg/L/day)	11.30 ± 0.20^a^	11.12 ± 0.20^a^	11.15 ± 0.17^a^	11.20 ± 0.21^a^
Total carotenoid (mg/L)	28.28 ± 1.85^b^	36.76 ± 1.04^a^	36.31 ± 0.53^a^	38.72 ± 1.04^a^
Specific total carotenoid (mg/L/day)	1.18 ± 0.08^b^	1.53 ± 0.04^a^	1.51 ± 0.02^a^	1.61 ± 0.04^a^
Percentage of total carotenoid per biomass	0.255 ± 0.019^b^	0.298 ± 0.015^a^	0.286 ± 0.011^a^	0.313 ± 0.018^a^
Lutein (mg/L)	1.025 ± 0.063^b^	1.672 ± 0.004^a^	1.652 ± 0.006^a^	1.889 ± 0.004^a^
Percentage of Lutein per total carotenoid	85.980 ± 0.492^b^	86.239 ± 0.460^b^	95.854 ± 0.470^a^	91.579 ± 0.390^ab^
β-carotene (mg/L)	0.035 ± 0.002^a^	0.030 ± 0.002^a^	0.039 ± 0.001^a^	0.044 ± 0.002^a^
Percentage of β-carotene per total carotenoid	2.664 ± 0.082^a^	1.836 ± 0.072^b^	2.151 ± 0.031^ab^	2.123 ± 0.068^ab^
Astaxanthin (mg/L)	0.010 ± 0.001^a^	0.011 ± 0.001^a^	0.012 ± 0.001^a^	0.015 ± 0.001^a^
Percentage of astaxanthin per total carotenoid	0.786 ± 0.078^b^	0.590 ± 0.035^a^	0.699 ± 0.020^ab^	0.745 ± 0.022^b^

a,b Different superscript letters within the same row indicate significant differences among treatments (p < 0.05, Tukey’s HSD test).

Chlorophyll dynamics (Fig. 5b) followed growth trends and nitrate availability. Concentrations increased sharply from days 4 to 9, with further gains between days 10 and 12 in CO_2_-supplemented cultures, suggesting enhanced photosynthetic capacity during recovery. Peak chlorophyll levels in the 3.0 % CO_2_ treatment support the hypothesis that elevated inorganic carbon boosts photosystem efficiency through increased Calvin cycle flux.^41^ Levels subsequently declined after day 16, consistent with the onset of senescence and nutrient exhaustion.

Carotenoid accumulation (Fig. 5c) remained modest during the early green stage (days 0–4) but accelerated after day 5 in CO_2_-supplemented cultures. By day 9, total carotenoid concentration in the 3.0 % CO_2_ group reached 1.60 ± 0.02 mg/L—12.5 % and 21.9 % higher than in the 1.0–2.0 % and control cultures, respectively. These findings were mirrored by carotenoid-to-biomass ratios (Fig. 5d), which consistently favored CO_2_-enriched treatments. Enhanced RuBisCO activity under elevated CO_2_ likely increased 3-phosphoglycerate (3PGA) availability, providing precursors for both fatty acid and carotenoid synthesis.^42^ Importantly, CO_2_ addition was restricted to the green stage to mitigate excessive acidification (pH < 4), which otherwise disrupts bicarbonate equilibrium and reduces growth. During the red stage, CO_2_ withdrawal coupled with salinity stress ensured continued carotenoid induction without compounding acid stress.

**Fig. 6:**
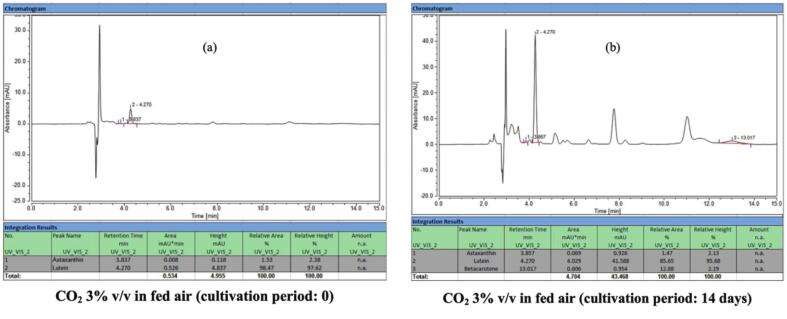
HPLC chromatograms illustrating the carotenoid profiles of *C. humicola* cultivated under 3 % v/v CO_2_ in fed air at (a) day 0 and (b) day 14. Major peaks correspond to astaxanthin (∼3.8 min), lutein (∼4.3 min), and β-carotene (∼13.1 min).

Over the full cultivation period (days 10–24), carotenoid accumulation was consistently higher in CO_2_-enriched groups, with total yields 29.98 %, 28.39 %, and 36.92 % greater than control under 1.0 %, 2.0 %, and 3.0 % CO_2_, respectively (Table 2). Comparable trends have been reported in *Chlorella zofingiensis* and *Haematococcus pluvialis*, where CO_2_ supplementation enhanced carbon flux toward isoprenoids.^43^, ^44^ HPLC chromatograms (Fig. 6) provided further resolution of pigment composition: lutein and astaxanthin dominated at baseline, but after 14 days of stress induction, β-carotene became the predominant pigment (84.02 %), accompanied by a 9-fold increase in total carotenoids. This metabolic plasticity illustrates *C. humicola*’s ability to redirect flux from the α-carotene branch (lutein) to the β-carotene branch under stress.

#### 3.2.4 Carotenoid composition and profiling

**Fig. 7:**
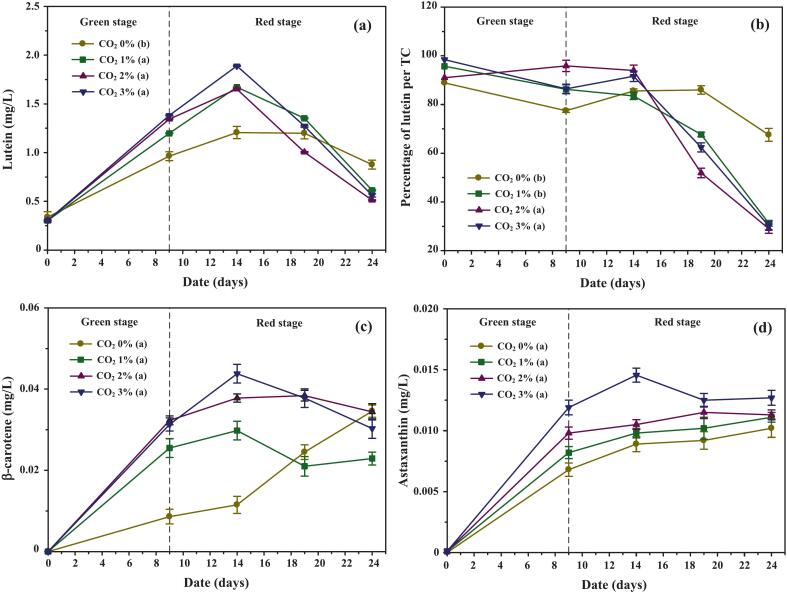
Concentrations and relative distribution of individual carotenoids in *C. humicola* cultured under varying CO_2_ concentrations (0 %, 1 %, 2 %, and 3 % v/v) during the green stage (days 0–9). Measured parameters include: (a) lutein concentration (mg/L), (b) lutein content as a percentage of total carotenoids (TC), (c) β-carotene concentration (mg/L), and (d) astaxanthin concentration (mg/L). Data are expressed as mean ± SE (n = 3). Different lowercase letters (a–b) denote statistically significant differences among CO_2_ treatments (Tukey’s HSD, p < 0.05). The vertical dashed line indicates the transition from the green to the red cultivation stage.

Temporal analysis of lutein, β-carotene, and astaxanthin during cultivation (Fig. 7a-d) revealed lutein as the predominant carotenoid throughout the experiment. In CO_2_-supplemented cultures, lutein concentration rose rapidly during the green stage, peaking at day 14, then declining toward day 24. In contrast, the control group showed slower initial accumulation, with levels converging by day 19 and eventually surpassing CO_2_-enriched treatments on day 24. This pattern reflects the dual impact of CO_2_: accelerated nitrate depletion and ROS-mediated antioxidant signaling stimulated early carotenoid synthesis, but prolonged stress in CO_2_ treatments exacerbated cell mortality and reduced lutein content later in the red stage. The marked decline in lutein proportion within total carotenoids (Fig. 7b) further supports this interpretation.

The β-carotene accumulation (Fig. 7c) followed a gradual upward trajectory in all groups, with the highest levels observed under 3 % CO_2_ supplementation. Peaks occurred around day 14, followed by modest declines by days 19–24. This decline may reflect metabolic channeling of β-carotene into downstream products such as astaxanthin, a phenomenon also documented in *Haematococcus pluvialis* under high-stress conditions.^45^, ^46^ Compared with *Scenedesmus obliquus*, which accumulates β-carotene under stress but at relatively lower levels, *C. humicola* demonstrated stronger β-carotene retention, highlighting its moderate stress tolerance.

Astaxanthin levels remained consistently low across treatments (Fig. 7d), increasing only slightly after day 14, with the maximum concentration detected in the 3 % CO_2_ treatment. Despite being a minor component overall, this modest induction aligns with reports that astaxanthin requires more severe or prolonged stress conditions for strong induction.^46^ Table 2 confirms that lutein dominated the carotenoid profile, with β-carotene and astaxanthin representing smaller fractions, in agreement with species-specific biosynthetic regulation.

One-way ANOVA confirmed that CO_2_ concentration exerted a significant effect on carotenoid biosynthesis (F[3176] = 177.6, p < 0.001, η2 = 0.942). Tukey’s HSD test showed that all CO_2_ treatments produced significantly higher carotenoid concentrations than the control (p < 0.01). The 3 % CO_2_ group achieved the highest total carotenoid concentration (38.72 ± 1.04 mg/L), representing a 36.9 % increase compared to the control (28.28 ± 1.85 mg/L, t = 8.52, p < 0.001). No significant differences were observed among 1 %, 2 %, and 3 % CO_2_ treatments, suggesting that concentrations above 1 % exert comparable enhancement effects.

These findings underscore *C. humicola*’s strong capacity for lutein production, supported by CO_2_-induced metabolic flux toward the xanthophyll pathway. The moderate but consistent increases in β-carotene and astaxanthin reflect species-specific responses, contrasting with *Dunaliella salina* and *Haematococcus pluvialis*, which preferentially accumulate β-carotene or astaxanthin under extreme conditions.^45^, ^46^, ^47^, ^48^ Thus, *C. humicola* is distinguished as a promising lutein-rich strain that can complement established carotenoid producers in biotechnological applications.

### 3.3 Effect of white light intensity on carotenoid production

#### 3.3.1 Growth response and cell viability

Light intensity strongly modulates microalgal growth and survival by influencing both photosynthetic efficiency and stress signaling pathways. During the red stage (days 10–24), *C. humicola* exhibited distinct responses to white light intensities ranging from 3,500 to 100,000 Lux.

**Fig. 8:**
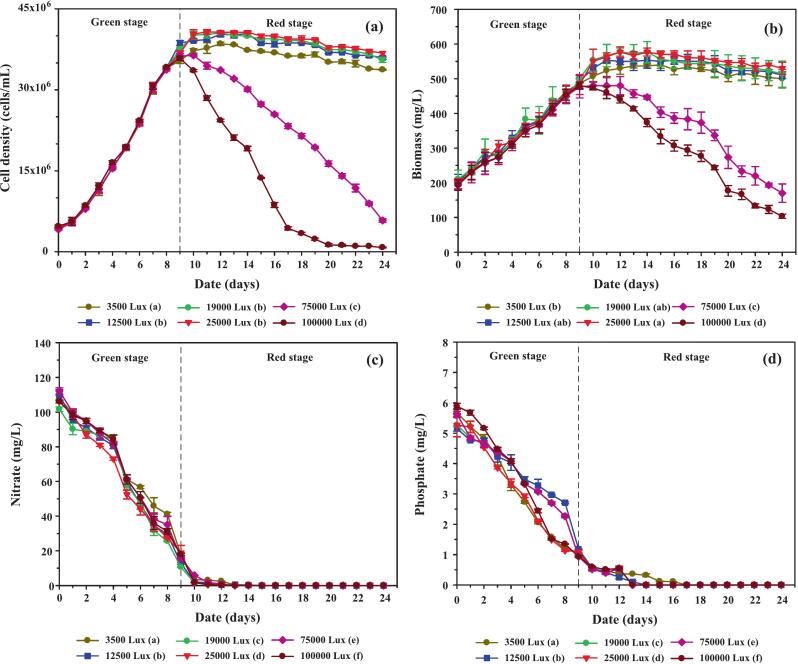
Growth performance, biomass accumulation, and nutrient uptake of *C. humicola* cultivated under different white light intensities (3,500, 12,500, 19,000, 25,000, 75,000, and 100,000 Lux) during the red stage (days 10–24). Parameters include: (a) cell density (cells/mL), (b) biomass concentration (mg/L), (c) nitrate concentration (mg/L), and (d) phosphate concentration (mg/L). Data are presented as mean ± SE (n = 3). Different lowercase letters (a–f) indicate statistically significant differences among treatments (Tukey’s HSD, p < 0.05). The vertical dashed line represents the transition point between the green and red cultivation stages.

As shown in Fig. 8a, cultures exposed to low light (3,500 Lux) showed minimal growth between days 10–13, followed by stable cell densities until day 24, indicating that low irradiance maintained viability but did not stimulate additional growth. Moderate light intensities (12,500–25,000 Lux) supported more robust growth, with final cell densities 6.55 ± 1.33 % higher than the 3,500 Lux group, suggesting that this range provided sufficient energy for carbon assimilation without inducing stress. In contrast, high irradiance (75,000–100,000 Lux) caused sharp declines in cell density, with cultures under 100,000 Lux falling below initial values, indicative of photoinhibition and oxidative damage.

Biomass patterns (Fig. 8b) closely mirrored cell density trends. Under moderate white light intensities, biomass remained relatively stable until day 14 before gradually declining, whereas under extreme irradiance (100,000 Lux), a continuous decrease was observed from day 10 onward, suggesting that cellular degradation exceeded growth. As shown in Table 3, biomass productivity under moderate light (12,500–25,000 Lux) was 43.45 % higher than under high-intensity light and 3.66 % greater than under low light, highlighting the efficiency of moderate irradiance. These results align with previous findings in other Chlorophyta species, where intermediate light intensities enhanced photosynthetic performance and pigment biosynthesis, while excessive light exposure triggered reactive oxygen species (ROS) formation, damaged the photosynthetic apparatus, and ultimately inhibited growth.^49^

**Table 3:** Bioproducts obtained from *C. humicola* cultivated under different white light intensities for 24 days of cultivation. Data are expressed as mean ± SD (n = 3). Different superscript letters denote significant differences among light treatments at p < 0.05 according to Tukey’s HSD test. One-way ANOVA revealed a significant effect on total carotenoid production (F[5,264] = 10.8, p < 0.001).

Light intensity (Lux)	3,500	12,500	19,000	25,000	75,000	100,000
Biomass (mg/L)	11,246.67 ± 72.82^b^	11,533.30 ± 74.62^ab^	11,696.70 ± 75.68^ab^	11,743.30 ± 75.98^a^	8,606.67 ± 55.68^c^	7,646.67 ± 49.47^d^
Specific biomass (mg/L/day)	468.61 ± 14.84^b^	480.56 ± 15.05^ab^	487.36 ± 26.48^ab^	489.31 ± 17.45^a^	358.61 ± 20.45^c^	318.61 ± 14.35^d^
Fatty acid (mg/L)	3,770.00 ± 56.55^a^	3,743.33 ± 56.15^a^	3,763.33 ± 56.45^a^	3,633.00 ± 54.50^a^	2,610.00 ± 39.15^b^	1,996.67 ± 29.95^c^
Specific fatty acid (mg/L/day)	157.08 ± 2.53^a^	155.97 ± 2.51^a^	156.81 ± 2.52^a^	151.40 ± 2.44^a^	108.75 ± 1.75^b^	83.19 ± 1.34^c^
Chlorophyll (mg/L)	271.28 ± 4.74^a^	267.62 ± 4.11^a^	268.88 ± 5.00^a^	255.43 ± 4.75^b^	207.08 ± 5.90^c^	177.44 ± 5.74^d^
Specific chlorophyll (mg/L/day)	11.30 ± 0.20^a^	11.15 ± 0.17^a^	11.20 ± 0.21^a^	10.64 ± 0.20^b^	8.63 ± 0.25^c^	7.39 ± 0.24^d^
Total carotenoid (mg/L)	28.282 ± 1.849^c^	29.850 ± 1.743^bc^	31.476 ± 1.266^b^	32.034 ± 1.518^a^	25.907 ± 1.128^d^	22.244 ± 2.031^e^
Specific total carotenoid (mg/L/day)	1.178 ± 0.077^c^	1.244 ± 0.073^bc^	1.323 ± 0.053^b^	1.346 ± 0.063^a^	1.079 ± 0.047^d^	0.927 ± 0.085^e^
Percentage of total carotenoid per biomass	0.245 ± 0.019^a^	0.250 ± 0.015^ab^	0.263 ± 0.020^b^	0.264 ± 0.017^b^	0.305 ± 0.027^c^	0.308 ± 0.034^c^
Lutein (mg/L)	1.025 ± 0.063^d^	1.487 ± 0.022^a^	1.524 ± 0.028^a^	1.488 ± 0.023^a^	1.205 ± 0.051^c^	1.033 ± 0.089^d^
Percentage of Lutein per total carotenoid	85.979 ± 2.145^c^	98.816 ± 2.000^a^	90.812 ± 0.510^b^	87.385 ± 2.500^c^	90.656 ± 0.051^b^	91.250 ± 3.100^b^
β-carotene (mg/L)	0.035 ± 0.002^c^	0.049 ± 0.002^b^	0.057 ± 0.003^a^	0.048 ± 0.002^b^	0.046 ± 0.004^b^	0.042 ± 0.003^bc^
Percentage of β-carotene per total carotenoid	2.664 ± 0.082^d^	3.344 ± 0.170^c^	3.603 ± 0.310^b^	2.836 ± 0.190^d^	5.077 ± 0.019^a^	3.906 ± 0.100^b^
Astaxanthin (mg/L)	0.010 ± 0.001^c^	0.004 ± 0.001^d^	0.025 ± 0.001^b^	0.019 ± 0.001^b^	0.039 ± 0.001^a^	0.040 ± 0.001^a^
Percentage of astaxanthin per total carotenoid	0.786 ± 0.090^c^	0.303 ± 0.070^d^	1.169 ± 0.070^b^	1.143 ± 0.070^b^	2.661 ± 0.070^a^	3.683 ± 0.080^a^

a,b,c,d,e Different superscript letters within the same row indicate significant differences among treatments (p < 0.05, Tukey’s HSD test).

#### 3.3.2 Nutrient utilization and conversion efficiency

Nutrient uptake dynamics closely reflected the interaction between light intensity and metabolic performance. As shown in Fig. 8c, nitrate consumption diverged after day 10. Under moderate light (12,500–25,000 Lux), nitrate was rapidly depleted between days 10–14, consistent with active assimilation to sustain chlorophyll synthesis and cell division. By contrast, nitrate persisted longer in low-light cultures (3,500 Lux) due to reduced metabolic demand, while uptake under high light (75,000–100,000 Lux) was markedly impaired despite abundant photon availability. This inhibition is likely attributable to photodamage and oxidative stress, which curtailed metabolic activity and accelerated cell mortality.

Phosphate utilization trends (Fig. 8d) further support this interpretation. In low-light cultures, phosphate remained detectable until day 17, suggesting slower but sustained ATP generation. Under moderate and high irradiance, phosphate depletion occurred earlier (by day 13), reflecting both accelerated metabolic turnover and stress-induced phosphate demand. However, the concomitant decline in biomass under 100,000 Lux indicates that phosphate assimilation alone could not counteract the loss of cellular integrity under severe light stress.

These observations highlight the dual role of irradiance as both a driver of growth and a source of physiological stress. Moderate light intensities supported efficient nutrient conversion while maintaining metabolic stability, whereas excessive irradiance disrupted photosystem II (PSII) function, enhanced reactive oxygen species (ROS) accumulation, and impaired carbon and nitrogen metabolism.^50^, ^51^

For large-scale photobioreactor applications, these findings underscore the importance of optimizing light delivery to balance stress induction for secondary metabolite biosynthesis with preservation of cell viability. In the case of *C. humicola*, white light intensities of 12,500–25,000 Lux during the red stage appear to offer the most favorable conditions for nutrient assimilation and subsequent carotenoid accumulation.

#### 3.3.3 Pigment and metabolite accumulation

**Fig. 9:**
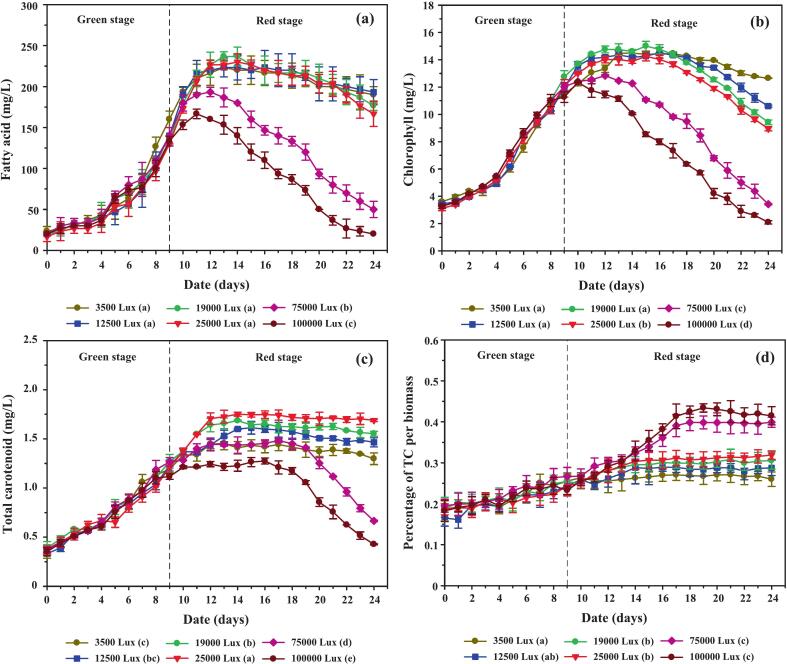
Biosynthesis of intracellular components in *C. humicola* cultured under various white light intensities (3,500, 12,500, 19,000, 25,000, 75,000, and 100,000 Lux) during the red stage (days 10–24). Measured parameters include: (a) fatty acid concentration (mg/L), (b) chlorophyll concentration (mg/L), (c) total carotenoid concentration (mg/L), and (d) percentage of total carotenoids (TC) per biomass. Data are expressed as mean ± SE (n = 3). Different lowercase letters (a–e) indicate statistically significant differences among treatments (Tukey’s HSD, p < 0.05). The vertical dashed line denotes the transition from the green to the red stage.

As shown in Fig. 9a, fatty acid accumulation was strongly influenced by nutrient limitation and salinity stress, with light intensity acting as a secondary factor. Under low (3,500 Lux) and moderate (12,500–25,000 Lux) light, fatty acid levels rose sharply between days 10–14, coinciding with nitrate depletion (Fig. 8c) and the first NaCl addition (100 mM). Thereafter, fatty acid concentrations declined as NaCl stress intensified (200–300 mM), reflecting reduced photosystem efficiency and increased cell mortality. Among these conditions, 25,000 Lux yielded the highest fatty acid content, indicating that moderate irradiance enhanced lipid metabolism without causing severe photodamage. In contrast, high light (75,000–100,000 Lux) triggered only a brief accumulation (days 10–12) followed by a sharp decline, suggesting that excessive irradiance induced rapid cell damage that outpaced lipid biosynthesis. Consistent with Table 3, overall fatty acid yield and specific productivity were markedly lower under high light, corroborating observations in *Scenedesmus obliquus* that extreme irradiance suppresses lipid accumulation despite stress induction^52^.

Chlorophyll dynamics (Fig. 9b) largely reflected photosynthetic performance. Moderate light enhanced chlorophyll content up to day 14, in line with higher cell densities (Fig. 8a). Beyond this point, concentrations declined more steeply than in low light cultures, likely due to photoinhibition and nitrogen reallocation from chlorophyll to stress-protective metabolites such as carotenoids and fatty acids. High light caused an immediate reduction in chlorophyll, consistent with accelerated cell death and photosystem degradation. Table 3 confirms that while accumulated and specific chlorophyll yields were reduced under high irradiance, the percentage of chlorophyll relative to biomass increased, reflecting disproportionate biomass loss. Such trade-offs have also been reported in *Haematococcus pluvialis*, where light stress triggered chlorophyll catabolism as part of a protective metabolic shift.^53^

Total carotenoid accumulation trends (Fig. 9c) further highlight the dual role of light as both a stimulus and a stressor. Cultures exposed to moderate irradiance accumulated the highest carotenoid levels, peaking at days 10–13 before declining in parallel with reduced viability. Higher irradiance (75,000–100,000 Lux) stimulated an initial increase but caused reduced overall productivity due to photodamage and salinity stress. Although carotenoid content per unit biomass increased at higher light (Fig. 9d), total yield and specific productivity declined, indicating that cell death outweighed biosynthetic benefits. Similar patterns have been reported in *H. pluvialis* and *Scenedesmus obliquus*, where excessive light accelerates reactive oxygen species (ROS) formation, leading to photoinhibition despite carotenoid induction.^54^, ^55^

Overall, the results demonstrate that moderate white light (12,500–25,000 Lux) optimally supports fatty acid, chlorophyll, and carotenoid accumulation in *C. humicola*. High irradiance enhances stress signals but compromises viability, underscoring the importance of carefully balancing light-driven induction of secondary metabolism with cellular stability in large-scale systems.

#### 3.3.4 Carotenoid composition and profiling

**Fig. 10:**
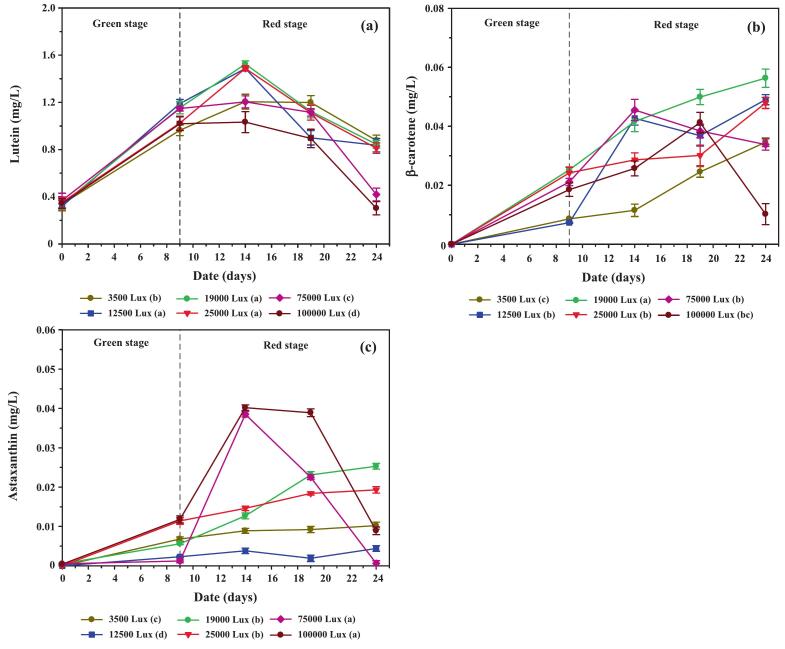
Concentrations of individual carotenoids in *C. humicola* cultivated under various white light intensities (3,500, 12,500, 19,000, 25,000, 75,000, and 100,000 Lux) during the red stage (10–24 days). (a) Lutein concentration (mg/L), (b) β-carotene concentration (mg/L), and (c) Astaxanthin concentration (mg/L). Values are expressed as mean ± SE (n = 3). Different lowercase letters (a–d) in the legend denote statistically significant differences among treatments (Tukey’s HSD, p < 0.05). The vertical dashed line represents the transition point from the green to the red stage.

The concentration profiles of lutein, β-carotene, and astaxanthin under varying white light intensities during the red stage are presented in Fig. 10. Lutein remained the predominant carotenoid across all treatments, accounting for >85 % of total pigments (Table 3). Under moderate light (12,500–25,000 Lux), lutein concentration peaked on day 14, then gradually declined as cell viability decreased. High-intensity light (75,000–100,000 Lux) suppressed lutein accumulation, with levels remaining low and relatively stable due to photoinhibition and severe cell mortality (Fig. 8a).

The β-carotene content increased steadily under low and moderate irradiance but declined under high light after day 14, consistent with reduced viable biomass. In contrast, astaxanthin was strongly induced under high light, peaking earlier (day 14) before declining sharply. Under moderate light, astaxanthin accumulation continued more gradually until day 24, suggesting that sustained but non-lethal stress favors secondary carotenoid biosynthesis.

These results indicate a stress-driven shift in pigment composition: from lutein dominance under mild conditions toward higher proportions of β-carotene and astaxanthin under intense light stress. Similar responses have been reported in *Haematococcus pluvialis* and *Scenedesmus obliquus*, where high irradiance (≥150 µmol photons m^−2^ s^−1^) stimulated astaxanthin accumulation but reduced overall yield due to photodamage.^56^, ^57^ Likewise, *Tetraselmis* sp. *CTP4* exhibited enhanced β-carotene and lutein at 170 µmol photons m^−2^ s^−1^ blue light, though productivity plateaued at higher intensities because of metabolic overload.^58^

Statistical analysis confirmed that light intensity significantly affected carotenoid productivity (F[5,264] = 10.8, *p* < 0.001, η^2^ = 0.868). Post-hoc comparisons revealed that 25,000 Lux yielded the highest carotenoid concentration (32.03 ± 1.52 mg/L), significantly greater than both low light (3500 Lux, 28.28 ± 1.85 mg/L, t = 2.72, *p* < 0.01) and high light (100,000 Lux, 22.24 ± 2.03 mg/L, t = 6.69, *p* < 0.001). Intensities above 75,000 Lux caused severe mortality, reducing productivity despite increases in astaxanthin percentage.

To further manipulate pigment composition, blue light (420–460 nm) was incorporated, targeting absorption peaks of chlorophyll *a* and b to enhance excitation of PSI and PSII. While this strategy stimulated β-carotene and astaxanthin as photoprotective metabolites, excessive excitation exacerbated ROS formation, amplifying oxidative stress.^59^, ^60^ Thus, spectral modulation must be carefully balanced to maximize secondary carotenoids without compromising viability.

Overall, these findings demonstrate that carotenoid biosynthesis in *C. humicola* is light-tunable: moderate white light optimizes total yield, whereas high light and blue light supplementation shift profiles toward stress-associated pigments such as β-carotene and astaxanthin. This tunability underscores the species’ potential for targeted, scalable production of specific carotenoids in photobioreactor systems.

### 3.4 Effect of blue light intensity on carotenoid production

#### 3.4.1 Growth response and cell viability

**Fig. 11:**
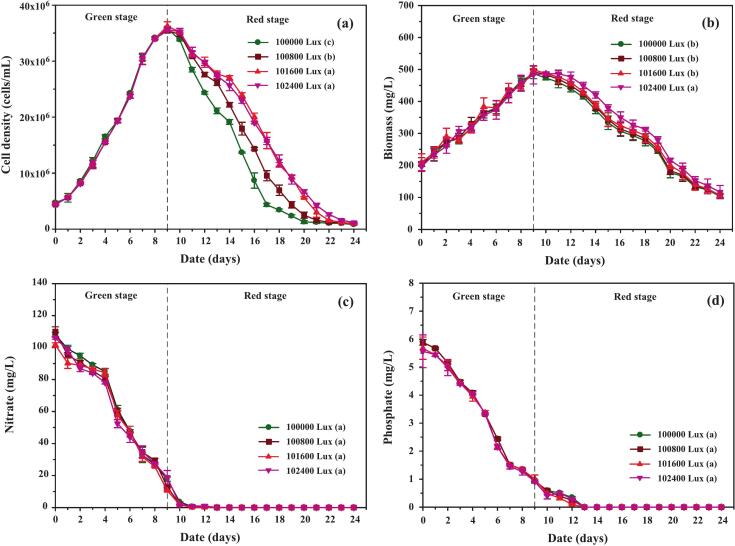
Growth performance, biomass accumulation, and nutrient uptake of *C. humicola* cultured under 100,000 Lux white light supplemented with blue light at 800, 1,600, and 2,400 Lux during the red stage (days 10–24). Panels show (a) cell density (cells/mL), (b) biomass concentration (mg/L), (c) nitrate concentration (mg/L), and (d) phosphate concentration (mg/L). Data are presented as mean ± SE (n = 3). Different lowercase letters (a–c) indicate significant differences among treatments (Tukey’s HSD, p < 0.05). The dashed vertical line marks the transition from the green to the red stage.

Blue LED light (800–2,400 Lux) was introduced to supplement 100,000 Lux white light, resulting in total irradiances of 100,800–102,400 Lux, while maintaining culture temperature at 35.0 ± 1.5 °C. As shown in Fig. 11a, cell density increased more under combined white and blue light than under white light alone, with the 1,600 and 2,400 Lux treatments yielding higher biomass between days 12 and 20. This enhancement is likely due to blue light absorption by carotenoids, which function as photoprotective agents. Specifically, lutein, β-carotene, and astaxanthin absorb within the blue spectrum at ∼444 nm, 466 nm, and 482 nm, respectively, thereby helping cells dissipate excess energy and mitigate photooxidative stress.^61^

Biomass accumulation trends (Fig. 11b) generally paralleled those of cell density, with only the 2,400 Lux blue light treatment exhibiting a modest increase relative to other conditions. These findings indicate that although blue light supplementation during the red stage enhances cell proliferation and viability, its impact on overall biomass accumulation remains limited. Instead, the primary role of blue light appears to involve the stimulation of carotenoid biosynthesis and the reinforcement of cellular photoprotection under high-light stress conditions.

#### 3.4.2 Nutrient utilization and conversion efficiency

Fig. 11c and d show that nitrate and phosphate depletion followed comparable trends across all blue light treatments, with complete exhaustion occurring during the red stage regardless of light intensity. This indicates that blue light supplementation did not markedly influence nutrient uptake rates. Instead, the observed differences in carotenoid production appear to be primarily driven by photoregulation rather than nutrient dynamics. In other words, while nitrogen and phosphorus availability constrained overall metabolic capacity, the modulation of light quality determined how carbon and energy fluxes were allocated toward pigment biosynthesis. These results suggest that the stimulatory effect of blue light on carotenoid accumulation is attributable to enhanced activation of light-dependent stress signaling pathways, rather than altered nutrient assimilation.^62^

#### 3.4.3 Pigment and metabolite accumulation

**Fig. 12:**
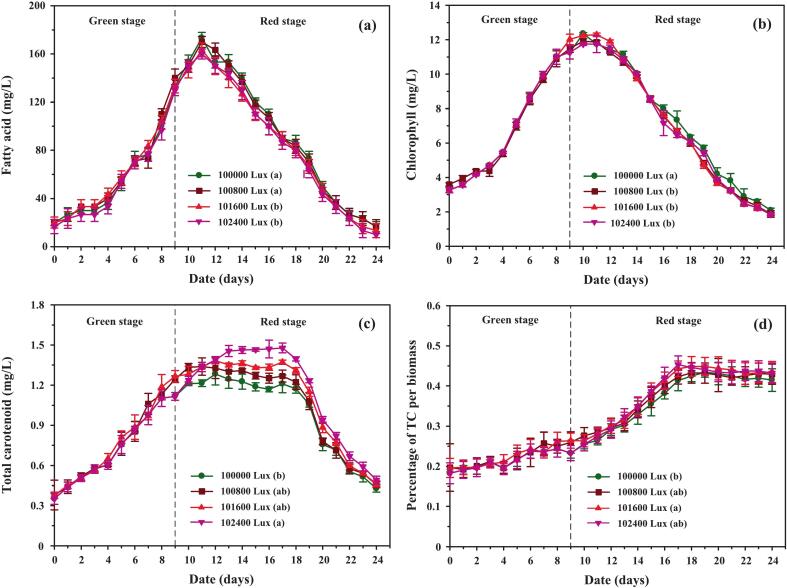
Intracellular accumulation of biochemical components in *C. humicola* cultivated under 100,000 Lux white light with supplemental blue light intensities of 800, 1,600, and 2,400 Lux during the red stage (days 10–24). Panels show (a) fatty acid concentration (mg/L), (b) chlorophyll concentration (mg/L), (c) total carotenoid concentration (mg/L), and (d) percentage of total carotenoids (TC) per biomass. Values represent mean ± SE (n = 3). Different lowercase letters (a–b) denote statistically significant differences among treatments (Tukey’s HSD, p < 0.05). The dashed vertical line indicates the transition from green to red stage.

Blue light supplementation substantially enhanced pigment and metabolite accumulation in *C. humicola*. Fatty acid levels (Fig. 12a) increased sharply between days 11 and 17, coinciding with intensified stress, before stabilizing across all treatments. Chlorophyll concentrations (Fig. 12b) declined more rapidly in cultures exposed to combined white–blue light compared to white light alone, reflecting blue-light-induced stress and the likely reallocation of nitrogen from chlorophyll biosynthesis toward carotenoid production.

Carotenoid accumulation was strongly stimulated under blue light (Fig. 12c). Among treatments, 2,400 Lux yielded the highest total carotenoid concentration, while the carotenoid-to-biomass ratio (Fig. 12d) also increased, reaching 5.70–11.19 % higher than white light alone (Table 4). This indicates that blue light acts as an effective photostress factor, intensifying ROS generation and photosystem excitation, which in turn stimulates flux through carotenoid biosynthetic pathways.

**Table 4:** Bioproducts accumulated by *C. humicola* cultivated under blue light supplementation for 24 days. Data are expressed as mean ± SD (n = 3). Different superscript letters indicate significant differences among treatments (*p* < 0.05, Tukey’s HSD test). One-way ANOVA indicated a significant effect of light intensity on total carotenoid production (F[3,104] = 14.1, *p* < 0.05).

Light intensity (Lux)	100,000	100,800	101,600	102,400
Total carotenoid (mg/L)	22.244 ± 2.031^b^	23.512 ± 1.623^ab^	24.141 ± 0.773^a^	24.734 ± 0.869^a^
Specific total carotenoid (mg/L/day)	0.927 ± 0.085^b^	0.973 ± 0.048^ab^	1.006 ± 0.032^a^	1.031 ± 0.036^a^
Percentage of total carotenoid per biomass	0.308 ± 0.034^b^	0.330 ± 0.026^ab^	0.336 ± 0.025^a^	0.331 ± 0.020^ab^
Lutein (mg/L)	1.033 ± 0.089^b^	1.133 ± 0.023^ab^	1.188 ± 0.032^a^	1.212 ± 0.031^a^
Percentage of lutein per total carotenoid	91.250 ± 0.590^a^	91.203 ± 0.230^a^	93.824 ± 0.320^a^	96.185 ± 0.450^a^
β-carotene (mg/L)	0.041 ± 0.003^d^	0.039 ± 0.002^c^	0.040 ± 0.002^b^	0.041 ± 0.002^a^
Percentage of β-carotene per total carotenoid	3.906 ± 0.100^c^	4.399 ± 0.180^b^	6.980 ± 0.280^a^	5.011 ± 0.340^b^
Astaxanthin (mg/L)	0.040 ± 0.001^a^	0.050 ± 0.001^a^	0.056 ± 0.003^a^	0.059 ± 0.002^a^
Percentage of astaxanthin per total carotenoid	3.683 ± 0.020^c^	4.399 ± 0.100^b^	4.853 ± 0.320^b^	5.011 ± 0.180^a^

a,b,c,d Different superscript letters within the same row indicate significant differences among treatments (p < 0.05, Tukey's HSD test).

These findings are consistent with earlier studies reporting similar responses in *Coelastrum* sp. and *Dunaliella salina*, where blue light exposure upregulated key carotenogenic genes such as phytoene synthase (PSY) and β-carotene ketolase (BKT), ultimately enhancing carotenoid accumulation.^63^ Collectively, the results underscore the role of spectral modulation in selectively regulating metabolic pathways, offering practical strategies for boosting carotenoid yields in controlled cultivation systems.

#### 3.4.4 Carotenoid composition and Profiling

**Fig. 13:**
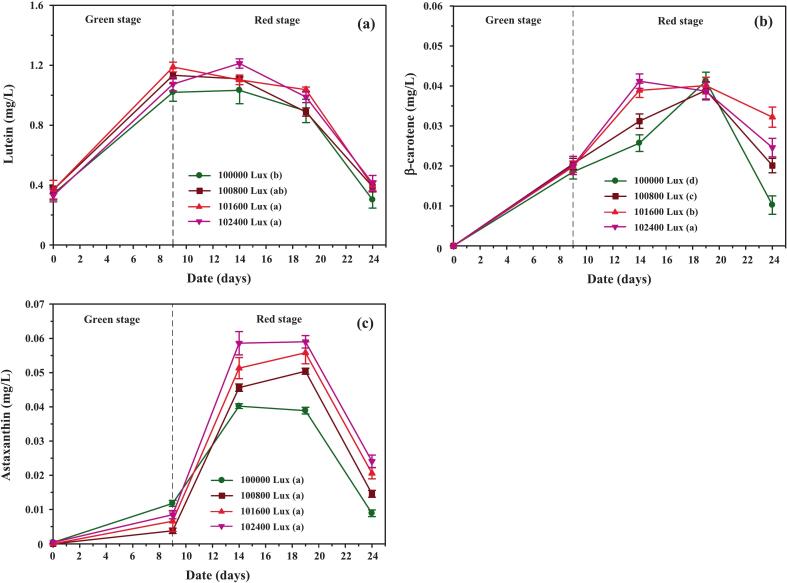
Carotenoid accumulation in *C. humicola* under 100,000 Lux white light and 800, 1,600, and 2,400 Lux blue light during the red stage (days 10–24). Shown are concentrations of (a) lutein, (b) β-carotene, and (c) astaxanthin (mg/L). Data are mean ± SE (n = 3). Different lowercase letters indicate significant differences among light treatments (Tukey’s HSD, p < 0.05). The dashed line marks the transition from green to red stage.

The concentration dynamics of β-carotene, astaxanthin, and lutein under varying blue light intensities are shown in Fig. 13a-c. All three carotenoids increased under combined white–blue light compared to white light alone, with the most pronounced effect observed in astaxanthin under 2,400 Lux blue light. This highlights the role of blue light as a potent inducer of secondary carotenoids, particularly in response to oxidative stress.

**Fig. 14:**
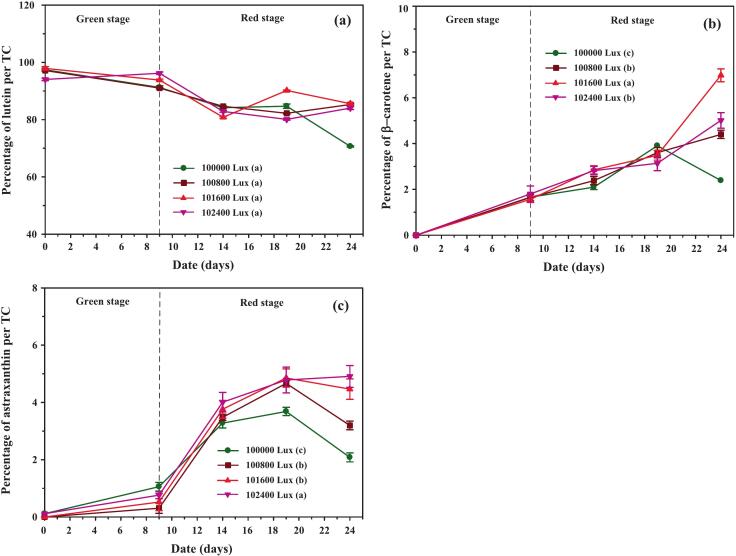
Distribution of individual carotenoids expressed as a percentage of total carotenoids (TC) in *C. humicola* cultured under white light (100,000 Lux) supplemented with different blue light intensities (800, 1,600, and 2,400 Lux) during the red stage (days 10–24). (a) Lutein (% of TC), (b) β-carotene (% of TC), and (c) astaxanthin (% of TC). Values represent mean ± SE (n = 3). Different lowercase letters (a–c) indicate statistically significant differences among treatments based on Tukey’s HSD test (p < 0.05). The dashed vertical line marks the transition from the green to the red stage.

Examining the percentage of lutein in the total carotenoids (Fig. 14a), a declining trend was observed as the cultivation period progressed in the red stage. However, by day 24, cultures receiving both white and blue light maintained higher lutein percentages than those exposed to white light alone. The percentage of β-carotene (Fig. 14b) increased steadily across all experimental conditions. However, a sharp decline occurred on day 24 in cultures exposed only to white light, likely due to high cell mortality between days 17 and 24 (Fig. 11a). The percentage of astaxanthin (Fig. 14c) showed that combining blue light with white light effectively stimulated astaxanthin production and accumulation within the cells throughout the red stage, a trend consistent with the astaxanthin concentration data in Fig. 13c.

These results are consistent with reports in *Haematococcus pluvialis* and *Chlorella zofingiensis*, where moderate blue or mixed-spectrum light enhanced astaxanthin accumulation, while excessive intensities (>90,000 Lux) reduced productivity due to PSII damage and photoinhibition.^64^ Similarly, our findings confirm that moderate supplementation with blue light enhances carotenoid diversification without compromising overall biomass stability.

Table 4 further confirms that lutein remained the dominant carotenoid under all conditions (>85 % of total carotenoids). However, β-carotene and astaxanthin were significantly upregulated by blue light supplementation, with maximal percentages of 6.98 ± 0.28 % and 5.01 ± 0.18 %, respectively. Statistical analysis supported these observations: one-way ANOVA revealed a significant enhancement of total carotenoid production by blue light (F[3104] = 14.1, *p* < 0.05, η^2^ = 0.682). The addition of 2400 Lux blue light to 100,000 Lux white light resulted in the highest total carotenoid yield (24.734 ± 0.829 mg/L), representing an 11.2 % increase relative to white light alone (22.244 ± 2.031 mg/L, t = 3.70, *p* < 0.05).

Taken together, these findings demonstrate that moderate blue light (1,600–2,400 Lux) effectively enhances the biosynthesis of high-value secondary carotenoids in *C. humicola*. Importantly, this induction occurs without excessive biomass loss, underscoring the potential of spectral light modulation as a practical strategy for targeted pigment production in large-scale photobioreactor systems.

### 3.5 Comparative evaluation and implications

The findings from this study underscore the potential of *C. humicola* for carotenoid biosynthesis under stress-induced batch cultivation. The optimal condition was achieved through 3.0 % CO_2_ enrichment during the green stage, followed by salinity and light stress in the red stage. Under this regime, the total carotenoid yield reached 38.72 ± 1.04 mg/L with a specific productivity of 1.61 ± 0.04 mg/L/day and carotenoid content of 0.313 ± 0.001 % per dry cell weight. Lutein remained the predominant pigment (91.579 ± 0.039 %), while β-carotene and astaxanthin constituted 2.123 ± 0.068 % and 0.745 ± 0.039 %, respectively.

Moderate CO_2_ enrichment and light modulation represent scalable interventions for industrial photobioreactors (PBRs). Commercial gas-mixing systems and programmable LED arrays can replicate these conditions with relatively low energy demand. Importantly, moderate CO_2_ levels reduce buffering requirements while still enhancing biomass and pigment yields. Coupled with waste-derived CO_2_ inputs and closed-loop PBRs, this approach aligns with sustainable bioprocessing and carbon mitigation strategies.

When compared to light-driven cultivation strategies, *C. humicola* exhibited distinct carotenoid responses. At 25,000 Lux white light, yields were slightly lower (32.03 ± 1.52 mg/L), with reduced lutein but higher β-carotene and astaxanthin fractions (2.836 ± 0.190 % and 1.143 ± 0.070 %). High-intensity white light (100,000 Lux) further enhanced β-carotene (3.906 ± 0.100 %) and astaxanthin (3.683 ± 0.080 %), but total yield declined due to cell mortality. Supplementing white light with blue LED (1600–2400 Lux) maximized β-carotene (6.980 ± 0.280 %) and astaxanthin (5.011 ± 0.180 %), illustrating the tunability of pigment profiles through spectral regulation. However, prolonged high-intensity exposure increased thermal and oxidative stress, underscoring the trade-off between pigment diversification and cellular integrity.

Comparisons with established microalgal species highlight the unique advantages of *C. humicola* (Table 5). *Scenedesmus obliquus* accumulated 8.01 mg/g biomass lutein under red light,^65^ while *Dunaliella salina* reached ∼120 mg/g biomass β-carotene under balanced red–blue light.^66^ These species favor single pigment dominance, unlike *C. humicola*, which supports co-accumulation of lutein, β-carotene, and astaxanthin. *Chlorella vulgaris* showed zeaxanthin and lutein enhancement under high-intensity flashing light but at the cost of chlorophyll degradation,^67^ whereas *C. humicola* maintained pigment stability under moderate stress. Similarly, *Tetraselmis* sp. CTP4 accumulated ∼3 mg/g each of lutein and β-carotene under blue LEDs,^68^ but required strong oxidative stress, while *C. humicola* responded effectively to more moderate inputs. Though *Scenedesmus falcatus* achieved extreme β-carotene yields (919.83 mg/g) under high irradiance,^69^ such conditions are difficult to scale sustainably.

**Table 5:** Comparison of Light Intensity Effects on Carotenoid Production in Various Microalgal Species.

Microalgal species	Light intensity & type	Maximum carotenoid yield	Carotenoid types enhanced	Notable observations	Reference
*Chlorococcum humicola*	White 25,000 Lux; Blue 2,400 Lux	32.03 mg/L (Total); 0.46 mg/L (Astaxanthin)	β-carotene, Astaxanthin	Moderate white light optimized total pigments; blue light enhanced secondary carotenoids	This study
*Scenedesmus obliquus*	LED lights (680 nm, 50–300 μmol photons/m^2^/s)	8.01 mg/g biomass	Lutein	LED light induced stress promoted carotenoid formation	^65^
*Dunaliella salina*	Red: Blue (660 nm:450 nm), 1:1, 300 μmol/m^2^/s	∼120 mg/g biomass	β-carotene	Red light enhanced cell division. Blue light stimulates β-carotene accumulation.	^66^
*Chlorella vulgaris*	Continuous light 200 μmol/m^2^/s with flashing light 7000 μmol/m^2^/s	Zeaxanthin increased 10-fold and lutein increased by ∼39 %	Zeaxanthin, Lutein	High light improved pigments accumulation but reduced chlorophyll *a* and b.	^67^
*Tetraselmis* sp. *CTP4*	Blue LED, 450 nm, 170 μmol/m^2^/s	3.17 mg/g biomass (Lutein), 3.21 mg/g biomass (β-carotene)	Lutein, β-carotene	Rapid light intensity induced pigments production for photooxidative damage.	^68^
*Scenedesmus falcatus*	Light, 100–1000 μmol/m^2^/s	∼919.83 mg/g (β-carotene), 34.56 mg/g (Lutein)	β-carotene, Lutein	Carotenoid biosynthesis positively correlated with light dose.	^69^

Taken together, these results position *C. humicola* not as the highest-yielding strain in absolute pigment content, but as a versatile platform for light-tunable, multi-carotenoid production. Its ability to balance yield, pigment diversity, and cell viability under moderate stress regimes makes it a strong candidate for industrial biorefineries focused on scalable, energy-efficient, and sustainable carotenoid production.

## 4 Conclusions

This study demonstrates the potential of *Chlorococcum humicola* TISTR 8551 for carotenoid production under controlled CO_2_ enrichment and light regimes in an air-lift photobioreactor. Cultivation with a nitrate-to-phosphate molar ratio of approximately 31:1 supported rapid growth and efficient nutrient utilization, while initiating the red stage on day 10 following nitrogen depletion enhanced carotenoid biosynthesis.

CO_2_ enrichment, particularly at 3 % v/v, significantly increased total carotenoid yield (38.72 ± 1.04 mg/L), with lutein as the dominant pigment. In contrast, high-intensity white light (25,000–100,000 Lux), especially when supplemented with low-intensity blue light (≤2,400 Lux), favored β-carotene and astaxanthin accumulation. However, prolonged exposure induced significant cell mortality.

Overall, optimal carotenoid production was achieved by balancing stress induction with cell viability—using moderate CO_2_ enrichment to enhance lutein and combining white–blue light for secondary carotenoids. These findings provide practical strategies for scalable and light-regulated carotenoid production in microalgal bioprocesses.

Comprehensive statistical analysis showed that all cultivation interventions produced significant effects with large effect sizes, underscoring their biological and practical relevance. Among the tested factors, CO_2_ enrichment had the strongest effect on carotenoid production (F[3176] = 177.6, *p* < 0.001, η^2^ = 0.942), followed by white light intensity optimization (F[5264] = 10.8, *p* < 0.001, η^2^ = 0.868) and blue light supplementation (F[3104] = 14.1, *p* < 0.05, η^2^ = 0.682). These results indicate that CO_2_ availability is the dominant driver of carotenoid biosynthesis in *C. humicola*. Light intensity and spectral quality further modulate the distribution of individual carotenoids. Collectively, these findings provide strong statistical support for the proposed cultivation strategies and their potential application in industrial-scale carotenoid production.

## CRediT authorship contribution statement

**Chatchai Kunyawut:** Supervision, Resources, Methodology, Investigation, Funding acquisition, Conceptualization. **Idtisak Paopo:** Validation, Supervision, Methodology, Formal analysis. **Chakkrit Umpuch:** Writing – review & editing, Writing – original draft, Visualization, Project administration, Data curation.

## Declaration of competing interest

The authors declare that they have no known competing financial interests or personal relationships that could have appeared to influence the work reported in this paper.
